# Nanowire-Based Biosensors: From Growth to Applications

**DOI:** 10.3390/mi9120679

**Published:** 2018-12-19

**Authors:** Pranav Ambhorkar, Zongjie Wang, Hyuongho Ko, Sangmin Lee, Kyo-in Koo, Keekyoung Kim, Dong-il (Dan) Cho

**Affiliations:** 1 School of Engineering, University of British Columbia, Kelowna, BC V1V 1V7, Canada; pambhorkar@yahoo.com; 2 Department of Electrical and Computer Engineering, Institute of Biomaterials and Biomedical Engineering, University of Toronto, Toronto, ON M5S 3M2, Canada; zongjie.wang@utoronto.ca; 3 Department of Electronics, Chungnam National University, Daejeon 34134, Korea; hhko@cnu.ac.kr; 4 Department of Biomedical Engineering, Kyung Hee University, Yongin 17104, Korea; sangmlee@khu.ac.kr; 5 Department of Biomedical Engineering, University of Ulsan, Ulsan 44610, Korea; kikoo@ulsan.ac.kr; 6 ASRI/ISRC, Department of Electrical and Computer Engineering, Seoul National University, Seoul 08826, Korea

**Keywords:** nanowire, sensor, biological applications, medical applications, nanomaterial

## Abstract

Over the past decade, synthesized nanomaterials, such as carbon nanotube, nanoparticle, quantum dot, and nanowire, have already made breakthroughs in various fields, including biomedical sensors. Enormous surface area-to-volume ratio of the nanomaterials increases sensitivity dramatically compared with macro-sized material. Herein we present a comprehensive review about the working principle and fabrication process of nanowire sensor. Moreover, its applications for the detection of biomarker, virus, and DNA, as well as for drug discovery, are reviewed. Recent advances including self-powering, reusability, sensitivity in high ionic strength solvent, and long-term stability are surveyed and highlighted as well. Nanowire is expected to lead significant improvement of biomedical sensor in the near future.

## 1. Introduction

Biomedical sensors with high sensitivity could make it possible to detect diseases in their early state, drastically increasing the chance of potentially life-saving detection and intervention. For example, when breast cancer is detected at an early stage (local disease) and treated with existing therapies, the five year survival rate is greater than 90%, but drops to around 20% upon developing into late stages (distant disease) [1]. Highly sensitive biosensors for the early detection of cancer are yet demanded [2,3]. Though much research has been carried out in an attempt to increase the sensitivity of biomedical sensors, recent developments in nanotechnology will provide the most promising solutions in the area of biomedical sensing [4,5]. Nanotechnology includes broad science and engineering research areas to study materials and structures less than approximately 100 nm [6]. In case of the nanometer scale, physical and chemical properties of materials are highly dependent on surface-to-volume ratios and quantum size effects, resulting in completely different properties than those at the macroscale [7]. 

Since the surface area-to-volume ratio is enormous at the nanoscale, it affects most of the regions on the sensing structure, making a nanostructure ultrasensitive to changes on its surface. The electron movement of the nanostructure is confined by quantization effects, resulting in quantum size effects, in which the discrete energy levels of the device depend on the size of the structure. In addition, both the excited energy of semiconductors at the lowest state and the strength of volume-normalized oscillator are increased by decreasing the scale [8], allowing the nanostructures to have a high energy conversion efficiency and relatively low thermal noise [9]. The size of biomolecules (~10 to ~100 nm), which is in the same range of synthetic nanostructures, also benefits the development of biomedical sensors using nanotechnology.

The primary task of biomedical sensors is to detect and characterize chemical and biological species, ranging from applications in disease diagnosis to drug discovery. Using nanomaterials (e.g., nanowires, carbon nanotubes, and nanoparticles) with distinct optical, magnetic, and electrical properties, those primary biomedical tasks can be achieved readily [10]. For examples, semiconductor crystalline nanoparticles were used to detect the labeled disease markers and other biological species [11,12], and colloidal gold was used in optical imaging and magnetic resonance [13,14]. In addition, semiconductor nanowires made it possible to detect various species electrically and without labels [15]. These nanowires were fabricated from semiconductor materials [16] and their surface can be readily modified to become sensitive to chemical and biological species [17,18]. Carbon nanotubes, which can also be employed in biomedical sensors, however, are synthesized by mixing semiconductor materials and metals, requiring further purification [19,20]. In addition, the binding protocols of a variety of analytes to nanotubes have not been well established [16,21]. Therefore, nanostructures fabricated by nanowires are the most suitable option for biomedical sensors that have high sensitivity, uniformity, reproducibility, and scalability with relatively simple fabrication process [22,23].

This review includes working principles of nanowire-based biomedical sensors and a variety of applications in biological and medical fields, updating our previous reports [24,25], which do not overlap the contents of this paper. We also discuss the advancement of long-term stability, sensitivity in solvents with high ionic strength, reusability, and self-powering, as those advancements significantly impact on overcoming the current limitations of nanowire-based biomedical sensors. 

## 2. Working Principle and Fabrication Process

### 2.1. Fundamentals of Nanowire Field-Effect Transistors

**Figure 1:**
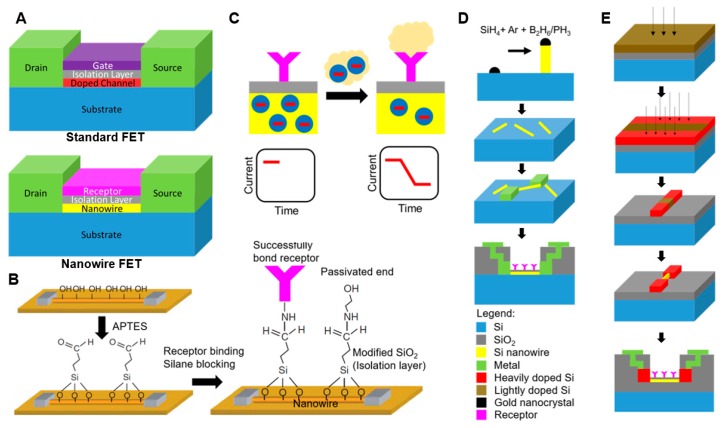
Conceptual overview of field effect transistors (FET) (adopted with permission from [24]). (**A**) Schematics illustrating the differences between standard and nanowire field-effect transistors. (**B**) An overview of the functionalization process of the nanowire (based on 3-aminopropoyltriethoxysilane (APTES)). The APTES converts the silicon–oxygen bonds into a silane layer, after which, it becomes possible to bind this layer with several receptors (adopted with permission from [31]). (**C**) Illustrates the operating principles of nanowire sensors. The charged molecules captured by the receptors induce variations in the conductivity of nanowire (there is either an increase or a decrease in the current that passes through it). The nanowire sensors fabricated using (**D**) the bottom-up approach and (**E**) the top-down approach.

Nanowire field-effect transistors (FETs) are a type of nanowire sensor that have originated from the standard planar FETs which consist of a gate, source, drain, and the body (Figure 1A). With a metallic material, the source and drain on the body are fabricated at the micro- or nanoscale. The gate, a critically thin isolation layer (e.g., SiO_2_), is fabricated between the source and drain, and generates electric potential variations to adjust the conductivity between the source and drain [26]. These variations in electric potential are usually in response to the application of an external voltage. Chemically or biologically charged species can also alter the potential and then conductivity by binding the charged species to the gate. This type of an electrical detection mechanism through an accumulation of charged species was proposed several decades ago [27]. However, it requires a lot of samples for detection due to the low sensitivity, preventing the use of the planar gate FET sensor in many applications.

In the nanowire FETs, doped channels and gates are replaced with nanowires and receptors, respectively (Figure 1A). Even though the structures of the FETs are different, the working principle of nanowire FETs remains the same as standard FETs in which the conductivity of nanowires (i.e., doped channels) is altered by external changes caused by charged species. Among various semiconductor nanowires that can be fabricated by silicon or other materials [28,29,30], silicon and silica nanowires are most widely used due to their high compatibility with the standard CMOS (complementary metal–oxide–semiconductor) technology. The natural growth of the oxide layer as the isolation layer on the silicon surface, and the easy modification of the silicon and silica surface, are other benefits of silicon and silica nanowires. Although the biomolecules have charges, the accumulation of biomolecules on the isolation layer does not alter the conductivity of nanowires since the original silicon and silica surface is not sensitive to biomolecules [14]. Therefore, it is necessary to make the surface functionalized and bound by receptors for sensing the specific charged species (e.g., DNA, RNA, viruses, etc.). By functionalizing the nanowire FETs, chemical connections between the surface of isolation layers coated on nanowires and biomolecules can be made (Figure 1B). Then, the receptors, which bind the collected species, induce an electric field onto the nanowires and alter the conductivity of nanowires. As we consider species as an input signal, the receptors play a similar role to the gate since they convert the input signal to changes in the conductivity of devices. Figure 1B shows a typical binding process using 3-aminopropoyltriethoxysilane (APTES), which is a type of receptor for DNA, peptide nucleic acid (PNA), and antibodies [16]. APTES converts silicon–oxygen bonds to a silane chemistry layer (Si–O–Si–X), where X can be modified further or linked with specific receptors. 

Figure 1C shows the working principle of the nanowire sensor. Upon the binding of the negatively charged species in an aqueous solution to the receptor, positive charges are induced on the Si nanowire FET surface. The generation of positive charges can either be considered as the disappearance of electrons (negatively charged carriers) or the appearance of holes (positively charged carriers), therefore changing the conductivity (or current) between the source and the drain. In the n-type Si nanowire, the conductivity will decrease as the charge carriers in this case are electrons. On the contrary, in p-type Si nanowires, the current will increase as the charge carriers in p-type nanowires are holes. A semiconductor parameter analyzer is used for the real-time monitoring of conductivity from the Si nanowire FETs [32,33]. Unlike the typical FETs on electronic circuit boards, the nanowire FET is usually in the ON state (i.e., DC currents always pass through). Also, since multiple nanowires are usually more sensitive to the accumulation of species and experience lower statistical noise as a result of averaging, an array of nanowires is commonly used in a single sensing device [34]. 

Surface charge variation of the isolation layer is another factor of changes in the conductivity of nanowires. The pH of solvents affects the surface charge of nanowires, resulting in the alteration of the conductivity of nanowires [14]. At low pH, the surface terminating in –NH_2_ group, as shown in Figure 1B, consumes positively charged holes from the nanowire surface and becomes –NH_3_^+^. At high pH, –SiOH consumes electrons from the nanowire surface and becomes –SiO–. The conductivity changes of nanowires due to pH is a combined effect of these two mechanisms [14]. The pH is an important factor to maintain the accuracy of nanowire sensors since measurement data from the same solutions with different pH will be inconsistent. 

Counterion condensation effect also contributes to the conductivity of nanowires. For instance, if the bound molecules are negatively charged in the solvent, they will be surrounded by positively charged counterions due to electrostatic interactions. Positive charges from counterions on a certain length are attracted to negative charges from the target biomolecules. As a result, charges from the target biomolecules are eliminated [35]. This length is called Debye length ($\lambda_{d}$). The Debye length is the measure of a charge carrier’s net electrostatic effect in a solution, which means how far those electrostatic effects are maintained. Therefore, the Debye length is critical for sensors to be able to differentiate the signal from target molecules that are in an electrostatic system. Over the Debye length, the negatively charged molecules become electrically neutral as the effect of negative charges is offset by positive charges coming from the electrostatic interaction of analytes, contributing to no changes in the conductivity of the nanowire. Therefore, only the target molecules within the Debye length contribute to the conductivity change. In addition, the detectability of negatively charged molecules decreases as the Debye length is shortened [35]. Long-enough Debye lengths can fully detect the charged molecules with high sensitivity. In the design of nanowire sensors, an optimal Debye length is a major parameter to be carefully considered, and a long Debye length ensures the counterions effect small [36]. The Debye length for the nanowire sensors can be approximately calculated as $\lambda_{D}\approx 0.304I^{-0.5}$, where *I* is the ionic strength of the solvent solution [37,38]. As in the equation, the Debye length gets increased as the ionic strength gets decreased. Thus, the sensitivity of nanowire sensor with a less conductive solvent is better than with the more conductive solvent. Significant increments in the Debye length was obtained by diluting the phosphate buffered saline (PBS) with the deionized water [39]. 

### 2.2. Fabrication Process

There are bottom-up and top-down methods that are utilized for the fabrication of nanowire sensors. As shown in Figure 1D, the bottom-up methods have been used to grow high-quality nanowires commonly on Si wafers [40]. Although most nanowires are cylindrical in shape, existing bottom-up techniques are capable of varying their cross-sectional shape, producing round, square, and triangular versions [41]. The fabrication process starts with growing Si nanowires using the chemical vapor deposition (CVD) method [31]. Si nanowires can be grown catalytically in the CVD reaction via the vapor–liquid–solid (VLS) mechanism [42]. Subsequently, the Si nanowires suspended in ethanol solution are deposited onto a silicon substrate. A photoresist is then spin-coated onto the substrate with deposited Si nanowires, and then the metal electrodes are patterned by the lift-off method process. The bottom-up fabrication ends with passivation and surface modification with receptor binding [31]. The isolation layer on the nanowire surface is easily achieved by exposing it to air or an oxygen environment. The bottom-up approach has a drawback of the randomly oriented nanowires, leading to the poor uniformity and low yield rate of nanowire sensors. In order to improve the orientation, additional alignment steps during fabrication are required, such as Langmuir–Blodgett [43], blown-bubble [44], microfluidic flow [45], contact printing [46], and electric-field [47,48]. Nevertheless, the standard CMOS fabrication process is not compatible with those alignment methods, making the mass production of the aligned nanowire sensors difficult. 

Unlike the bottom-up methods, the top-down methods, based on the microfabrication process on a silicon-on-insulator (SOI) wafer or a single-crystalline silicon (SCS) wafer, can fabricate aligned nanowires that are compatible with the standard CMOS technology. As shown Figure 1E, the fabrication process starts from doping low-density boron or phosphorous on the top Si layer of a SOI wafer. Subsequently, heavy density doping on the patterned area is conducted to define the source and drain leads, followed by forming micrometer-sized source and drain electrodes using reactive ion etching (RIE). Then, electron-beam (E-beam) lithography is used to fabricate nanometer-sized Si nanowires and the metal contact leads are formed by a thermal evaporation. Finally, the fabrication process ends with passivation and surface modification, same as the bottom-up method [49,50]. While the top-down methods are highly compatible with the CMOS processes with well-oriented nanowires, the diameter of nanowires is bigger than nanowires produced via the bottom-up method. Despite differences in nanowire orientation and materials for drain and source, these devices share the same architecture (e.g., drain/nanowire/source).

**Figure 2:**
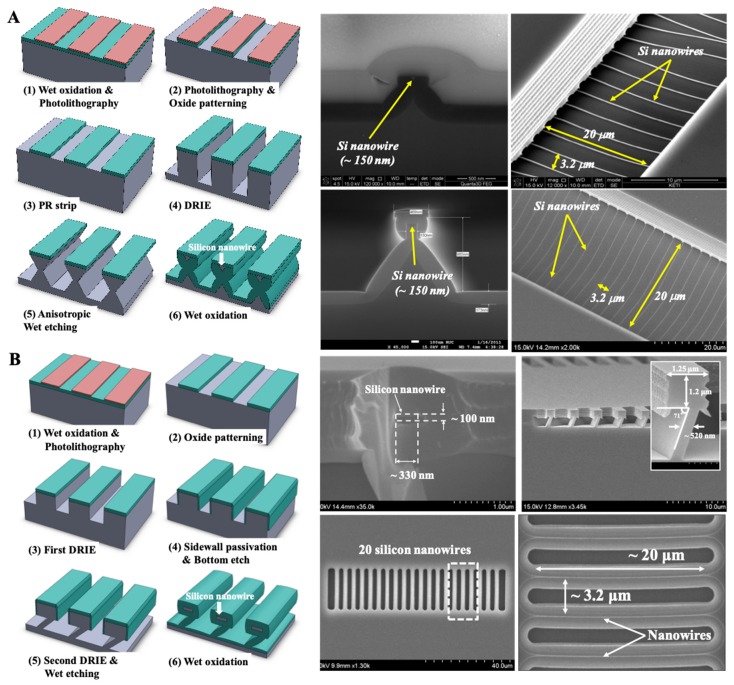
Top-down fabrication process and results using single-crystalline silicon (SCS) wafer. (**A**) (100)-silicon (adopted with permission from [51,52]). (**B**) (111)-silicon (adopted with permission from [53]).

Another silicon nanowire fabrication process using a top-down process based on bulk SCS wafer is shown in Figure 2. Generally, the fabrication is performed with combined processes of typical semiconductor manufacturing process, which uses photolithography, silicon dry etching, anisotropic wet etching, and thermal oxidation [51,52,53]. In these top-down fabrication methods, the width of the silicon nanowires is controlled by the thermal wet oxidation time. Its result is organized silicon nanowires. The schematics of the fabrication processes of (100)- and (111)-oriented SCS wafer are illustrated in Figure 2A,B respectively. For the (100)-silicon fabrication process, a 1000-Å-thick silicon dioxide is grown on the boron-doped silicon substrate using a thermal oxidation process. After photolithographic definition of the space and line, the silicon dioxide at the top layer is etched by the dielectric etching process. Then, photoresists are removed, and cleaning processes are performed. Then, the silicon deep RIE etching process is performed to define the rectangular-structured columns, where the silicon nanowires are located. Subsequently, anisotropic silicon wet etching process, using tetramethylammonium hydroxide (TMAH) solution, is carried on until the {111}-plane of the silicon surface is exposed. As shown in the Figure 2A, the hourglass-shaped silicon structures are fabricated, exposing the etch-stop {111}-surfaces. The arrayed silicon nanowire structures are fabricated by a thermal oxidation process, in which the upper layers of the hourglass structures are the silicon nanowires, and are isolated by the thermal oxide between the upper and lower layer. The height of the nanowire is determined by the photolithographic pattern width, which is an inherent property of the SCS wafer. The top-down (111)-silicon nanowire fabrication also starts with the thermal oxide growth process. Then, photolithography and dielectric etching processes are performed to define the line and space patterns. The primary deep silicon RIE process is performed for defining the height of the silicon nanowire. For (111)-silicon nanowires, the height and the width of the structures are defined separately, which can result in various structures, such as wires and ribbons. Then, thermal oxidation process is performed for passivation of the sidewalls of the whole structures. The plasma-enhanced anisotropic dielectric dry etching process is performed to expose the bottom surface of the structure, and secondary deep silicon RIE process is performed to fabricate the sacrificial layer. Subsequently, an anisotropic silicon wet etching process by TMAH solution is followed to reveal the {111}-planes. Finally, the thermal oxidation process is performed to fabricate the arrayed rectangular nanowire structures, after the passivated silicon dioxide layers’ removal.

One of critical components of the nanowire sensors is the fluid exchange system. The fluid exchange system delivers analytes or fluids to, or close to, the surface of the nanowire sensor. Polydimethylsiloxane (PDMS)-based channel devices are widely used for feeding analytes to nanowire sensors. In biosensing applications, PDMS is also advantageous due to its high biocompatibility [54,55]. However, the PDMS-based microfluidic fluid exchange system has a few limitations. Since fluid flow in microscale channels is characterized by laminar flow, particles inside the microfluidic channels are difficult to approach the surface of the nanowire sensors. In addition, there is the possibility of sensitivity reduction of the sensor as PDMS can absorb biomolecules [54]. In order to overcome these limitations of PDMS microfluidic channels, an acrylic chamber as a fluid exchange system has been developed [56]. 

## 3. Detection of Various Biological Agents

### 3.1. Proteins

Several new biomarkers have been discovered through research in proteomics and genomics that could potentially be used for diagnosing diseases [1]. In order to diagnose complicated diseases like cancer, that are characterized by considerable heterogeneity, single marker tests do not yield acceptable diagnoses [3]. It is important, therefore, to consider multiple biomarkers [6]. This is especially important for the treatment of cancer, where the detection of multiple biomarkers could determine the stage of the disease [6]. The first application of p-type silicon nanowire sensors was to detect proteins in a solution, electrically [16]. Since then, various nanowire and nanowire array-based platforms have been developed for simultaneously detecting multiple disease marker proteins [28,33,57,58].

Many contemporary studies have converged on developing platforms that can detect these disease markers real-time [59], as well as directly from whole blood [60]. It is also possible now to detect a wider variety of biomarkers like cardiac biomarkers [56]. Recent work has also demonstrated the application of nanostructured sensing platforms in detecting many proteins, label free, making it possible to diagnose pregnancy and diseases like diabetes, Parkinson’s disease, atherothrombosis, and autoinflammatory diseases [61]. Furthermore, recently, nanowire-on-a-chip platforms have been developed for the comprehensive detection of biomolecules, wherein nanowires carry out preconcentration, separation, filtering, and detection, enabling rapid and practical clinical use [62].

**Figure 3:**
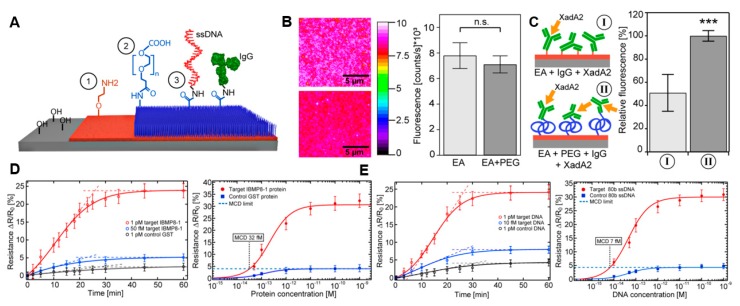
Generalized strategy for ultrasensitive detection of protein and DNA using nanowires (adopted with permission from [62]). (**A**) Schematic representing the functionalization process to allow high sensitivity. (1) Coupling ethanolamine to the surface; (2) attaching PEG cross-linker; (3) binding of biomolecules by peptide-binding. (**B**) False-colored image of fluorophore antibodies bound to the ethanolamine and PEGylated surfaces. No statistical difference was found on the amount of fluorescence. (**C**) PEGylated surface has higher relative fluorescence. The schematic illustrated the spacing introduced by PEG significantly improved the binding efficiency of antibodies. (**D,E**) Detection of protein antigen (**D**) and ssDNA (**E**) using the developed devices. The LOD is 6 fM and 1 fM for protein and ssDNA, respectively.

Janissen et al. presented comprehensive work that characterizes the effects of surface functionalization and covalent immobilization for protein detection from pathogens. In the study, surface functionalization with APTES, ethanolamine, was compared to the surface passivation via poly(ethylene glycol) (PEG) (Figure 3A). It was found that, compared to APTES, applying ethanolamine significantly promoted the bioreceptor density and coating homogeneity (Figure 3B,C). In addition, applying PEG led to many benefits, including (1) higher ligand binding specificity due to the reduction in non-specific adhesion; (2) increased receptor/ligand binding based on spatial separation; (3) additional spatial mobility for immobilized antibody, which further improves the antigen binding; and (4) minimized Debye length for sensing. They further applied the optimized functionalization strategy to detect a Chagas disease protein biomarker, IBMP8-1. The limit of detection (LOD) and minimal concentration detection are 5.7 fM and 32 fM, respectively, in serum (Figure 3D). The linear sensitivity region ranges from 90 to 500 fM. This article incorporates many recent strategies together and provides an improved methodology to fabricate robust, reliable, and sensitive protein biosensors using nanowires in a multiplexing format. 

### 3.2. DNA and RNA

It possible to detect specific sequences of DNA and RNA with nanowire sensors [63]. The surfaces of silicon nanowires consist of single-stranded sequences of PNAs that are placed to act as receptors for DNA [64]. The capability of silicon nanowires to detect DNA at the 10 femtomolar level has been demonstrated [65]. This is significantly better for DNA detection as compared to the quartz-crystal microbalance [66], surface plasmon resonance (SPR) [67] and nanoparticle-enhanced SPR [68].

The strategy introduced by Janissen et al. can also be applied to further improve the specificity and sensitivity of nanowire sensors [62]. Preliminary data revealed that the LOD of ssDNA detection of around 1 femtomolar level is achievable [62] (Figure 3E). In addition to the detection of individual strands of DNA, nanowires can also be utilized to detect the bonding between protein and DNA [38,69,70].

Similarly, nanowire devices can become RNA sensors by functionalizing PNA or DNA. For example, Lu et al. presented a way to fabricate a Si nanowire biosensor via anisotropic wet etching with self-limitation of Si<111> using tetramethylammonium hydroxide (TMAH) [71]. The self-limitation dramatically improved the uniformity of devices and lowered the manufacturing cost. This low-cost biosensor demonstrated a rapid detection of miR-21 and miR-205, two cancer-associated miRNAs, with the low limit of detection (LOD) down to 1 zeptomole. It also demonstrated great selectivity by clearly identifying the difference between target miRNA and a single-nucleotide mismatched sequence, as well as performing proper detection for spiked serum samples. In addition, nanowire biosensors have also been applied to detect virus gene in a label-free fashion. Huang et al. reported a strategy to amplify the detected signal via exonuclease III-assisted target recycling and achieved a LOD of 3.6 pM [72].

The capability of monitoring various cancer biomarkers in the DNA level, such as telomerase and carcinoembryonic antigen, opens a door for the use of nanowire-based DNA sensors for cancer diagnosis and treatment [33,73]. Recently, Yasui et al. unveiled massive number of cancer-related urinary microRNA candidates (~1000 types) with the help of multiplexed nanowires, covering not only the urologic malignancies (i.e., bladder and prostate) but also for other cancers (lung, pancreas, and liver). This provides a foundation for long-term work aiming to setup urine-based checkups for cancer [74]. However, in addition to this study, to date, the level of multiplexing is still below the demand for clinical needs. Simultaneous detection of multiple types of analytes from the same specimen holds the key to providing valuable data for downstream analysis and diagnostics [75]. The demand for high-throughput multiplexing has become even more critical when combined with machine learning-based diagnostics as it requires a large amount of data for proper training and accurate detection [75].

### 3.3. Viruses

The effective detection of viruses is one of the most important tasks of nanowire sensors for keeping our societies healthy and safe, since most viruses cause serious human diseases and can be used as biological weapons [76,77]. In recent years, Si nanowire sensors have been used to detect many dangerous viruses, including Dengue [78], influenza A H3N2 [79], H1N1 [80], and HIV [81]. The nanowire sensor surface is functionalized with antibodies that specifically bind to the target viruses, affecting the conductivity of the nanowire. For instance, Shen et al. developed a Si nanowire-based biosensor that could detect as many as 29 flu viruses/µL from exhaled breath condensate (EBC) samples [79]. Recently, Ibarlucea et al. introduced Si nanowires to detect Ebola VP40 matrix protein [82]. The limit of detection (LOD) was seen to be around 6.25 nM after 30 min incubation, outperforming the ELISA technique by six orders. With advantages including rapidness, accuracy, and portability, nanowire biosensors will play an increasingly important role in the point-of-care diagnostics of epidemic diseases.

## 4. Recent Advances in Nanowire Biosensors

This section provides a brief introduction to the important technologies that have emerged in recent years to solve the intrinsic problems of traditional nanowire-based biosensors, including in vivo sensing, integration with low-cost portable devices, and new strategies to process measured signals. With the help of these technologies, nanowire-based biosensors have become more practical and suitable for various biomedical applications.

### 4.1. In Vivo Sensing

**Figure 4:**
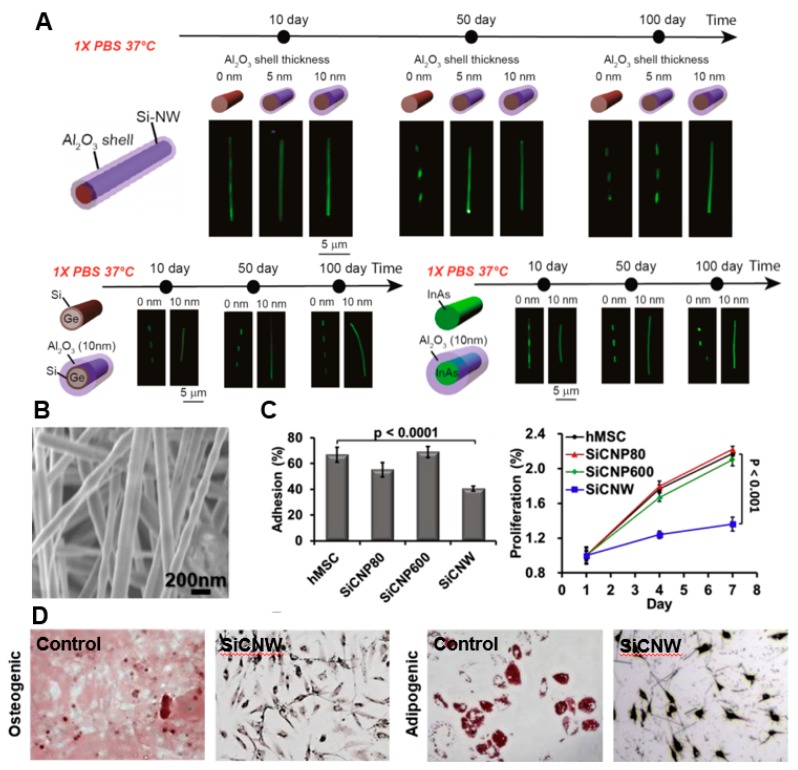
Design nanowires for in vivo sensing. (**A**) Improving long-term stability by Al_2_O_3_ shell coating (adopted with permission from [89]). (**B**–**D**) Cytotoxic effects of SiC nanowires to cell behavior and differentiation (adopted with permission from [89]). (**B**) SEM pictures of SiC nanowires. (**C**) Quantification of adhesion and proliferation of hMSCs on nanowires and nanoparticles. (**D**) Quantification of differentiation potency of hMSCs on SiC nanowires. Alizarin Red S and Oil Red O stain was utilized to quantify the differentiation toward osteogenic and adipogenic lineage, respectively.

The traditional applications of nanowires are mostly in vitro. However, recently, more work has been conducted in investigating sensors that can provide a continuous measurement of biosignals in vivo. Pilot studies by the Lieber group have demonstrated the possibility of injecting the nanowire sensors into human body for long-term recording of bioelectric signals, such as neural activities in the brain [83,84]. The in vivo environment presents a lot of challenges to the properties of nanowires. On the one hand, the in vivo microenvironment contains many ions that can dissolve the passivation layer (silicon oxidation) of silicon nanowires which naturally exist in air [85,86]. Early studies have reported the limited stability of nanowires upon being used with cells [87,88]. Zhou et al. recently introduced a coating method to enhance the long-term stability of nanowires [89]. Upon studying the protection of the 10 nm-thick Al_2_O_3_ shell, it was observed that the diameter of the nanowire remained almost the same for at least 100 days in 1× PBS at 37 °C, respectively, while the nanowire without the shell disappeared (Figure 4A). This coating strategy worked not only for Si nanowire, but also for Si–Ge complex and InAs complex. However, the increment of shell thickness downregulated the sensitivity of nanowire [90]. Hence, a trade-off must be made between performance and long-term stability. Regardless, this strategy can significantly improve the long-term stability of the nanowires in complex environments, opening up the further investigation of using nanowire as a platform for in vivo injectable electronics [91]. On the other hand, cells can physically sense the nanowires in vivo, leading to certain concerns of nanowires’ cytotoxicity and their effects on cell behavior. So far, the cell response to nanomaterials has not yet been systematically examined. Some preliminary studies indicate that nanowires do not significantly affect cell viability and proliferation [92]. On the contrary, more studies suggest that 1D nanostructures have a certain level of impact on cellular behavior, including cell viability, elongation, and differentiation [93]. For instance, Chen et al. reported a study on the toxicity of silicon carbide (SiC) nanowires, as shown in Figure 4B, and nanospheres on human mesenchymal stem cells (hMSCs) and cancer cells [94]. The impact of toxicity on metabolism, viability, proliferation, migration, oxidative stress, and differentiation potency were comprehensively examined. Interestingly, it was found that SiC nanowires are toxic to hMSCs, but not to breast cancer cell line MCF-7. The presence SiC nanowires, 200 nm in diameter, were seen to significantly reduce the adhesion and proliferation of hMSCs (Figure 4C) and to hamper their differentiation potency toward osteoblasts and adipocytes (Figure 4D). The toxicity is applied through the stress from altered morphology by nanowires, as proven by the significant changes of cytokine genes. Similarly, Alaraby et al. found that the Ni nanowire can cause DNA damage, gene alteration, and oxidative stress to cells, although the authors did not find a direct correlation between these negative effects to cytotoxicity and mutagenesis [95]. In summary, existing results suggest that the interaction between nanowires and cells is very sophisticated. It is, therefore, important for more research to be carried out in order to systematically understand and prevent the potential toxicity of nanowires during in vivo applications. 

### 4.2. Integration with Paper-Based Devices

**Figure 5:**
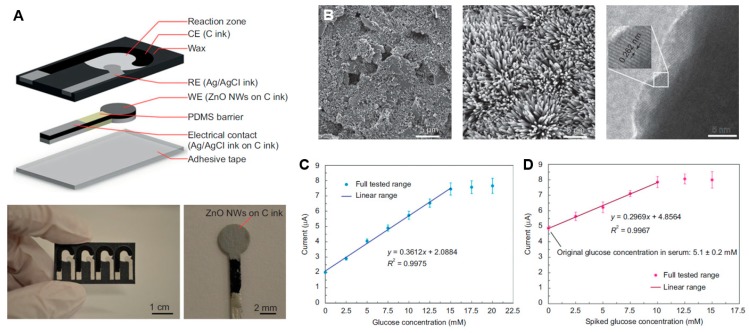
Integrate nanowires with disposable devices (adopted with permission from [97]). (**A**) Schematic and photograph of paper-based analytical devices (PADs) with ZnO nanowires. (**B**) Characterization of ZnO nanowires. From left to right: (1) SEM image of rough carbon surface before the growth of nanowire; (2) SEM image of nanostructured carbon surface with deposited ZnO nanowires; (3) TEM image for quantification of nanowires. (**C**) Calibration of sensor (output current vs. glucose concentration) in buffer. (**D**) Quantification of detected limits and linear range of the PADs in serum.

In addition to the traditional channel-based microfluidic devices, nanowires have recently been integrated with paper-based analytical devices (PADs) in order to facilitate biomarker detection in a portable fashion. The main advantages of PADs include low material and fabrication costs, as well as good biodegradability, as compared to silicon-based chips [96]. Li et al. reported a pioneering study using zinc oxide (ZnO) nanowires in PADs for glucose detection [97]. They printed the whole PADs using a conductive ink (i.e., carbon ink) and used it as a substrate to grow ZnO nanowires from deposited ZnO nanoparticle (Figure 5A). The ZnO nanowires were grown in a low-temperature (~70 °C) hydrothermal manner. The hydrothermal method is compatible with mass-production setups and exhibits good control over the size of ZnO nanowires (300 ± 50 nm, Figure 5B). This may facilitate several downstream applications. The authors then demonstrated the application of this sensor by detecting glucose level. The sensor was found to have a LOD of 94.7 µM and a linear range of up to 15 mM (Figure 5C,D). Although this is not comparable to some traditional sensors that can go all the way down to a LOD of 0.5 µM, this sensor holds great potential for applications in remote biosensing. However, there are many critical issues that need to be addressed before implementation. Firstly, the detection of many proteins relies on the presence of enzymes. Preserving enzymes at room temperature for a long time is a key challenge needed to be addressed for the successful implementation of PADs with nanowires. Secondly, to have a smaller LOD, the high surface roughness that significantly hampers detection at low concentrations in PADs must be addressed (Figure 5B). Nanofibrillated cellulose (NFC) paper [98,99] has recently come up as a solution to provide nanometer level surface roughness that could potentially improve the LOD of ZnO PADs. Lastly, to fulfill the demand for remote sensing, it is important to make these devices wireless and power-free. This demand can be met by the self-powering technology discussed in the following section.

### 4.3. Self-Powering

The self-powering strategies can be divided on to two categories, those using triboelectric nanogenerators and others using biofuels. Since its discovery in 2006, the piezoelectric effect of ZnO nanowires has been widely applied to generate power for various devices [100]. In principle, by applying a mechanical force to a well-aligned, serially connected ZnO nanowire array, a 1.26-volt output voltage has been achieved, which is enough to power the nanowire-based pH sensor [101] and the wireless data transmission system [102]. However, the mechanical energy generated by significant physical motion is mostly limited to lung, muscle, and cardiac tissues in vivo [103,104]. Hence, biofuels are a more popular power source in vivo. Researchers have also examined glucose-mediated strategies like glucose/O_2_ [105,106,107] and glucose/air for in vivo powering [108]. Additionally, Hansen et al. have demonstrated the potential of using a combination of piezoelectric effects and biofuels to build a self-powered system in vivo [109]. On the one hand, poly(vinylidene fluoride) nanofibers were utilized to harvest mechanical energy generated by the lung and heart (i.e., the breathing motion of the lung and the beating motion of the heart) while, on the other hand, biochemical energy was collected by a flexible enzymatic biofuel cell from biofluids. The synergetic effects of these two strategies lead to a higher output power and, potentially, a longer operating time.

### 4.4. Signal Processing and Data Analysis

**Figure 6:**
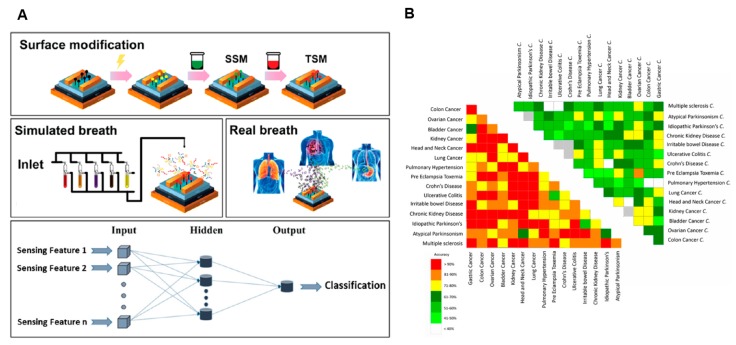
Signal processing strategy for multivariable nanowire biosensors. (**A**) Disease diagnostics based on machine learning: multivariable nanowire sensors were utilized to provide input for artificial neural networks (adopted with permission from [112]). (**B**) Big data strategy to correlate exhaled molecules detection by nanowire sensors with specific disease (adopted with permission from [113]).

Disease diagnostics usually involve the detection and analysis of multiple markers. Although there are many multiplexing biosensors being proposed, the majority of the research is still in the proof-of-concept stage, where the characterization of devices using spiked samples remains the gold standard. To fully claim the advantages of multivariable and multiplexing biosensors for practical diagnostics, signal processing techniques must be introduced to extract useful diagnostic information from the detected values. The idea of using signal processing for more meaningful data from nanowire sensor array originated from work in gas sensing [110]. For example, Cho et al. used a nanowire sensor array that consists of platinum, copper, indium, and nickel to detect vapors from explosive precursors including acetone, nitrobenzene, nitrotoluene, and octane [111]. They applied decision tree learning to classify the signals from the sensor array to predict the concentration of explosive precursors and access the risk of explosion with an accuracy beyond 90% and an error rate of less than 1%. In 2016, Shehada et al. brought this concept to diagnostic applications by using an array of Si nanowires to detect the disease breathprints of cancers [112] (Figure 6A). In this study, they first fabricated functionalized the Si nanowires by a single-step or a two-step modification. The array was then characterized by a simulated breath from a mixture of eleven disease markers linked with gastric cancer (GC), lung cancer (LC), asthma, and chronic obstructive pulmonary disease (AC). Upon successful characterization, an artificial neural network (ANN) was built, where the sensing features are the inputs of the model, and the sample classification label is the output. The ANN was then trained by a dataset with sensing features and a known disease state under examination. The trained ANN, together with the array of nanowire biosensors, was applied to analyze the real patient breath (*n* = 374). It was found that the array with ANN could distinguish almost all binary comparison of disease with an accuracy higher than 80%. However, the potential of machine learning is not fully realized in this study as the sample size is still limited, which results in poor accuracy rate (i.e., ~60%) for specific diseases with limited samples. Following the study, a clinical big dataset of the exhaled molecules for 17 diseases was generated from 1404 subjects measured by nanobiosensors [113] (Figure 6B). This study revealed 13 new volatile organic compounds that were associated with certain diseases. The success of these clinical studies has demonstrated a great model to translate the potential of nanobiosensors from proof-of-concept experiments to practical clinical diagnostics. In addition, with advances in unsupervised deep learning [114], more information could be extracted from the arrayed data in an automated but accurate fashion.

## 5. Summary and Perspective

With specific receptors, nanowire sensors have shown great potential to become a practical detection platform in biological and medical applications. The presented devices have several advantages, such as real-time transduction to electrical signal with high sensitivity and feasibility of label-free detection, as summarized in Table 1. In spite of these attractive features, some improvements are still required for commercialization. Even though its sensitivity is impressively high compared with other methods, its analytical signal intensity is still too low to be contaminated by high background noise, especially seen with in vivo environments. Improvements in receptor binding methods could resolve this higher sensitivity issue as well as more simple fabrication processing issues. In addition, a higher yield ratio of the current top-down fabrication methods enables a lower cost of commercialized products. Nevertheless, the success of a nanowire sensor will depend on how advanced it is compared with the current gold standards, such as PCR and ELISA, in terms of simplicity, sensitivity, specificity, and reliability. In particular, nanowire is expected to result in the development of promising wearable biosensors [115,116,117,118,119].

## Figures and Tables

**Table 1:** Summary of the salient features and the target application of nanowire-based biosensors.

Features	Application	Reference
Top-down fabrication process using SCS wafer	Photodiode and FET for the retinal prosthetic systems	[52,53]
High sensitivity using PEG cross-linker	Detection of protein and DNA	[62]
Long-term stability using Al_2_O_3_ shell coating	In vivo sensing	[89]
Integrating nanowires with disposable device	Glucose detection	[97]
Multivariable detection using machine learning	Multiple disease diagnosis	[112,113]
